# Comment on ‘Statin use and all-cancer survival: prospective results from the Women’s Health Initiative’

**DOI:** 10.1038/bjc.2016.395

**Published:** 2016-12-06

**Authors:** Luca Mascitelli, Mark R Goldstein

**Affiliations:** 1 Comando Brigata Alpina ‘Julia’/Multinational Land Force, Medical Service, 8 Via S. Agostino, Udine 33100, Italy; 2 NCH Physician Group, 1845 Veterans Park Drive, Suite 110, Naples, FL 34109, USA

**Sir**,

In a prospective cohort study of postmenopausal women with cancer (Wang *et al*, 2016), it was found that current use of statins and other cholesterol-lowering medication was associated with lower risk of cancer deaths. However, a dose-response relationship was not found, thus suggesting that the improved survival in patients with cancer could in fact be related to the higher baseline cholesterol, which in turn led to cholesterol-lowering drug prescription.

Baseline total cholesterol concentration of statin-treated populations is often higher than those of the general population, especially in the primary prevention settings (Thompson *et al*, 2008). Of note, in the study by Wang *et al* (2016), most of the enrolled women with current statin use (87%) had no coronary heart disease before cancer diagnosis.

Mounting evidence has established that low plasma levels of low-density lipoprotein cholesterol are associated with an increased risk of future cancer (Benn *et al*, 2011). Furthermore, in a long-term follow-up study, moderate total serum cholesterol was found to have a protective effect on 40-year cancer mortality (Panagiotakos *et al*, 2005), and an analysis of large statin randomised controlled trials demonstrated an inverse association between on-treatment low-density lipoprotein cholesterol levels and incident cancer (Alsheikh-Ali *et al*, 2008).

Increasing evidence has found that statins also exert their action beyond cholesterol reduction (pleiotropic effects), and a growing interest has been paid in their immunomodulatory effect, which may promote atherosclerotic plaque stability, but also hinder the host anti-tumour immune response, therefore, increasing cancer risk in certain segments of population (Goldstein *et al*, 2009). In particular, it appears that statins increase the risk of cancer in the elderly, who are more likely to harbour cancer cells because of their advanced age and associated immunosenescence (Gruver *et al*, 2007). In the Prospective Study of Pravastatin in the Elderly at Risk (PROSPER; The PROSPER Study Group, 2002), cancer incidence was increased significantly in subjects randomised to pravastatin over the 3.2-year study period. The mean age at trial entry was 75 years, and the decrease in cardiovascular disease mortality was offset by an equal increase in cancer mortality, resulting in unchanged overall mortality. Notably, the main age of postmenopausal statin users enrolled in the study by Wang *et al* (2016) was only 63.6 years.

Therefore, although the protective effect of higher cholesterol levels on cancer risk may not completely represent an etiologic link, the study by Wang *et al* (2016) suggests that current statin use might have selected the healthy statin user or unselected the unhealthy cancer patients with low cholesterol.

## References

[bib1] Alsheikh-Ali AA, Trikalinos TA, Kent DM, Karas RH (2008) Statins, low-density lipoprotein cholesterol, and risk of cancer. J Am Coll Cardiol 52(14): 1141–1147. 18804740 10.1016/j.jacc.2008.06.037

[bib2] Benn M, Tybjærg-Hansen A, Stender S, Frikke-Schmidt R, Nordestgaard BG (2011) Low-density lipoprotein cholesterol and the risk of cancer: a mendelian randomization study. J Natl Cancer Inst 103(6): 508–519. 21285406 10.1093/jnci/djr008

[bib3] Goldstein MR, Mascitelli L, Pezzetta F (2009) The double-edged sword of statin immunomodulation. Int J Cardiol 135(1): 128–130. 18485499 10.1016/j.ijcard.2008.01.023

[bib4] Gruver AL, Hudson LL, Sempowski GD (2007) Immunosenescence of ageing. J Pathol 211(2): 144–156. 17200946 10.1002/path.2104 PMC1931833

[bib5] Panagiotakos DB, Pitsavos C, Polychronopoulos E, Chrysohoou C, Menotti A, Dontas A, Stefanadis C (2005) Total cholesterol and body mass index in relation to 40-year cancer mortality (the Corfu cohort of the seven countries study). Cancer Epidemiol Biomarkers Prev 14(7): 1797–1801. 16030119 10.1158/1055-9965.EPI-04-0907

[bib6] The PROSPER Study Group (2002) Pravastatin in elderly individuals at risk of vascular disease (PROSPER): a randomised controlled trial. Lancet 360(9346): 1623–1630. 12457784 10.1016/s0140-6736(02)11600-x

[bib7] Thompson R, O’Regan C, Morant S, Phillips B, Ong S (2008) Measurement of baseline total cholesterol: new data from The Health Improvement Network (THIN) database. Prim Care Cardiovasc J 1(2): 107–111.

[bib8] Wang A, Aragaki AK, Tang JY, Kurian AW, Manson JE, Chlebowski RT, Simon M, Desai P, Wassertheil-Smoller S, Liu S, Kritchevsky S, Wakelee HA, Stefanick ML (2016) Statin use and all-cancer survival: prospective results from the Women’s Health Initiative. Br J Cancer 115(1): 129–135. 27280630 10.1038/bjc.2016.149 PMC4931370

